# Early end-effector-based gait training in non-ambulatory patients with visuospatial neglect after subacute stroke

**DOI:** 10.3389/fneur.2025.1639659

**Published:** 2025-10-01

**Authors:** Anna Gorsler, Doreen Ernst, Ulrike Grittner, Daniel Harnack, Peter Koßmehl, Jan Mehrholz, Carina Mueske, Philipp Schneider, Nadine Kuelzow

**Affiliations:** ^1^Kliniken Beelitz GmbH, Fachklinik für Neurologische Frührehabilitation, Beelitz, Germany; ^2^Faculty of Health Sciences Brandenburg, Brandenburg Medical School Theodor Fontane, Brandenburg, Germany; ^3^Institute of Biometry and Clinical Epidemiology, Charité-Universitätsmedizin Berlin, Berlin, Germany; ^4^Berlin Institute of Health, Berlin, Germany; ^5^Department of Public Health, Dresden University of Technology, Dresden, Germany; ^6^School of Health, Education and Social Sciences, University of Applied Health Science, Gera, Germany

**Keywords:** subacute stroke, visuospatial neglect, walking ability, end-effector-based gait training, rehabilitation

## Abstract

**Introduction:**

Early gait training plays a critical role in stroke rehabilitation, as reflected in relevant guidelines. However, patients with visuospatial neglect—a factor that negatively impacts gait recovery—have traditionally been excluded from robot-assisted gait training studies. To address this issue, our study examined the effects of end-effector-based gait training on subacute stroke patients with visuospatial neglect.

**Methods:**

A total of 43 patients were randomized in a controlled, assessor-blinded study and assigned either to end-effector-based gait training plus standard physical therapy or early verticalization with a standing frame plus standard physical therapy. All patients underwent nine training sessions over 2 weeks. We analyzed the primary outcome measure, the Functional Ambulation Category, using an ordinal regression model, reporting results for both the intention-to-treat population and the per-protocol sample, and also assessed trunk stability and balance as secondary outcomes.

**Results:**

Neither the intention-to-treat analysis (odds ratio [95% confidence interval]: 1.20 [0.30–4.78]) nor the per-protocol analysis (odds ratio: 4.08 [0.80–20.87]) revealed any significant overall superiority of gait training compared to standing training. However, the per-protocol analysis showed a promising pattern: Severely affected patients were more likely to improve their walking ability after gait training depending on their baseline Functional Ambulation Category score. Gait training also led to greater improvements in trunk stability and balance than standing training did.

**Conclusion:**

These results suggest that early adjunctive end-effector-based gait training could benefit a subgroup of severely affected, non-ambulatory, subacute stroke patients with visuospatial neglect.

**Clinical trial registration:**

DRKS00021654, www.drks.de/search/de/trial/DRKS00021654.

## 1 Introduction

Walking ability is a primary goal of stroke rehabilitation, given its essential role in regaining functional independence and improving quality of life (1, 2). Current clinical guidelines, such as the German ReMoS guideline, recommend starting high-intensity gait training early on in patients who are unable to walk during the subacute phase of stroke recovery (3, 4).

Visuospatial neglect (VSN) is a common consequence of a major stroke affecting the right hemisphere, affecting up to 38% of patients, typically alongside contralateral hemiparesis (5, 6). VSN poses a significant challenge to the rehabilitation process (7–10), as patients are frequently unaware of their deficits (11). VSN is a negative predictor of stroke outcomes (12–15). However, many studies on post-stroke gait rehabilitation have excluded patients with VSN (16), while those focusing on this group have addressed interventions specific to neglect (17–19). Consequently, evidence on the most effective gait rehabilitation strategies for patients with VSN after stroke remains limited.

Therefore, our study aimed to address the lack of research in this area by evaluating whether patients with severe impairment and an inability to walk (non-ambulatory patients) with VSN in the subacute phase after stroke could benefit from end-effector-based gait training (GT) in addition to standard therapy, compared with verticalization training in a standing frame (ST). We hypothesized that early GT, in addition to standard physical therapy, would be more effective than ST in restoring walking ability and improving trunk function and balance.

## 2 Methods

### 2.1 Study population

The study was approved by the local Ethics Committee (State Medical Association Brandenburg; S 13(bB)/2020), registered on 6 May 2020 at the trial register www.drks.de,^1^ and conducted in accordance with the Declaration of Helsinki. Prior to participation, informed consent was obtained from all patients. Patients with right hemispheric stroke and left-sided VSN were screened and recruited during their inpatient rehabilitation stay at Kliniken Beelitz GmbH (Brandenburg, Germany).

Our inclusion criteria for this study were as follows:

- At least 18 years of age
- Within the early subacute phase (7–80 days) post stroke
- First-ever right-hemispheric stroke (ischemic or hemorrhagic)
- Hemodynamically stable in standing (15 min in a standing frame)
- Unable to walk, as defined by the Functional Ambulation Category (FAC) score ≤ 2 (20, 21)
- A pathologically tilted subjective visual vertical (SVV > 2°) (22)
- Presence of VSN symptoms in at least one standard neglect test (see below).

Our exclusion criteria for this study were as follows:

- Previous stroke
- Severe traumatic brain injury
- Insufficient vigilance
- Severe substance abuse (alcohol/drugs)
- Any diagnosed psychiatric disorder
- Cognitive or communicative deficits affecting the capacity to consent
- Contraindications to end-effector-based gait training (e.g., with electromechanical gait trainers) such as severe osteoporosis, unstable fractures, mechanical ventilation, or open wounds around the trunk.

### 2.2 Design and procedure

A prospective, randomized controlled trial was conducted to demonstrate the initial efficacy of GT in patients with VSN (proof of concept). Participants were randomized using stratified block randomization with a fixed block size of 10 to ensure balanced allocation within strata. Stratification was based on baseline Trunk Impairment Scale (TIS) scores (low: ≤ 8; moderate: > 8) (23). Sealed opaque envelopes containing group assignments were used for allocation concealment and randomization and were distributed in a box. These envelopes were provided by someone not involved in the study and were only opened by the therapists responsible for treatment after the baseline assessments had been completed. Due to the nature of the intervention, neither the participants nor the therapists could be blinded to group allocation. However, blinded assessors used standardized protocols to evaluate the primary and secondary outcomes before and after the intervention. Sample size was estimated by PASS (Power Analysis and Sample Size Software) to test for differences in the primary outcome (walking ability measured by FAC) using an ordinal regression model. A 1.5:1 randomization ratio (GT: ST) of 25 to 18 provides 80% power to detect an odds ratio of 5.4 at a two-sided significance level (alpha) of 0.05.

### 2.3 Interventions

All patients received supplementary interventions in addition to their individual rehabilitation programs. Given the poor health status of the sample and the proof-of-concept nature of the study, we opted for a moderate training regimen. The supplementary training consisted of three sessions per week, each lasting 30 min, over a period of 3 weeks (total training time: 270 min). Electromechanical devices from the same manufacturer were used for interventions. The ST group received training in supported verticalization using a standing frame (THERA-Trainer balo, Germany). The GT group received gait training using an end-effector trainer (THERA-Trainer Lyra, Germany) at a minimum speed of 1.5 km/h and body weight support (BWS) up to 30%, enabling task-specific training (Supplementary Table 1). Training parameters were adopted from previous GT studies (16, 24, 25). Since no best-practice protocol existed for this cohort, treating therapists were allowed to make adjustments: in case of overexertion, BWS could first be increased up to 60%, followed by a reduction in walking speed. Specific weight-bearing metrics, which would have required brief independent standing, were not assessed, as this was largely not feasible in our cohort (26). Both interventions involved verticalization, orthostatic activation, and strengthening of the trunk and lower limb muscles. Patients in both groups also continued to receive their routine motor and neglect therapy. Neither group was permitted to receive the other’s specific training. After each training session, patients rated their perceived exertion on the Borg scale (27).

### 2.4 Outcome measurements

#### 2.4.1 Primary outcome

Walking ability was assessed using the Functional Ambulation Category (FAC), a reliable, valid, and recommended assessment tool (20, 21, 28). The FAC rates the level of assistance required during walking on a six-point scale:

- FAC 0 = unable to walk,
- FAC 1 = requires continuous manual support from one person to support weight and maintain balance,
- FAC 2 = needs continuous or intermittent light touch for balance/coordination,
- FAC 3 = independently on a level surface, but needs standby supervision,
- FAC 4 = independent walking, but requires assistance for stairs, inclines/uneven surfaces,
- FAC 5 = independent in all aspects of walking.

#### 2.4.2 Secondary outcomes

1. Trunk Impairment Scale (TIS) (23): Assesses static and dynamic sitting balance and trunk control. Scores range from 0 (no trunk control) to 23 (normal function).
2. Berg Balance Scale (BBS, 7-item short form) (29, 30): Assesses standing balance. Scores range from 0 (no balance) to 28 (normal balance).
3. Functional Reach Test (FRT) (31, 32): Assesses the maximal forward reach while sitting to evaluate balance and trunk control in a sitting position. The distance is measured in centimeters.

#### 2.4.3 Neglect, SVV, and mood

- VSN was assessed using standardized paper-and-pencil tests: the Bells Cancellation Test (33), Line Bisection, and Figure Copy Task (both from the German Behavioral Inattention Test battery (34)). A computerized “Saccade Position” test (Eyemove© Program (35)) was also administered.
- Catherine Bergego Scale (CBS) (36): A 10-item observational, performance-based checklist assessing impairment in activities of daily living caused by neglect.
- Bucket Test (37): A simple clinical method to assess potential shifts in subjective visual vertical (SVV). Patients, seated upright, look at the bottom of a bucket while the examiner rotates it from a random angle (six trials). The patient indicates when the line inside appears vertical. The deviation angle between the perceived and true vertical is measured; deviations > 2° indicate a spatial orientation deficit.
- Mood was assessed using the State–Trait-Anxiety-Depression Inventory (STADI, trait version) (38), a 20-item self-report questionnaire measuring general disposition toward anxiety and depression on a 4-point Likert scale, with higher scores indicating greater severity.

### 2.5 Statistical analysis

#### 2.5.1 Primary analysis: primary outcome walking ability (measured by FAC)

The analysis was conducted in accordance with the intention-to-treat (ITT) principle, comprising all randomized participants within their assigned groups. The management of missing outcome data involved the implementation of multiple imputation (30 datasets), a statistical method for handling missing data, via the R package “mice” (39). This approach involved the creation of imputation models that incorporated baseline FAC and TIS scores, sociodemographic variables, and outcomes. The effect estimates were then aggregated using Rubin’s rules (40). Given the potential for the primary analysis (ITT) to underestimate true treatment efficacy (41) by accounting for participants with minimal or no intervention exposure, a per-protocol (PP) sensitivity analysis was also performed. This analysis comprised all participants who completed ≥ 6 sessions (> 60%) with available post-training data. To estimate the treatment effect after training, the FAC scores were analyzed using proportional odds ordinal logistic regression. The model incorporated the training group (GT vs. ST reference), baseline FAC score (ordinal: 0, 1, 2), baseline TIS score, and the amount (minutes) of routine motor therapy as fixed effects. Subsequent to the observation of differential response patterns across baseline walking ability levels, a training group × baseline FAC interaction term was incorporated *post-hoc*. Baseline TIS was incorporated as the randomization stratification variable, while the minutes of routine motor therapy received were integrated into the analysis to control for concurrent interventions.

The regression model can be expressed as: logit[P(FAC_post ≤ k)] = αk + β₁(Group_GT) + β₂(FAC_baseline) + β₃(TIS_baseline) + β₄(Therapy_minutes) + β₅(Group_GT × FAC_baseline) where k represents the FAC category thresholds, αk are the intercepts for each threshold, and β coefficients represent the log-odds effects. The proportional odds assumption was verified using the Brant test (42). Marginal odds ratios with 95% confidence intervals were calculated for each baseline FAC level.

#### 2.5.2 Secondary analyses

We analyzed secondary outcomes (motor functions), performance on neglect tests, and mood after training using ANCOVA. The training group was used as the independent variable, with baseline scores, baseline TIS, and amount of routine motor therapy included as covariates. Training implementation was assessed using independent t-tests between the groups. An exploratory correlational analysis examined the association between baseline trunk stability and improvement in walking ability, without adjusting for multiple comparisons.

We used SPSS version 29 and R version 4.3.1 (43) with the packages brant, ordinal, tableone, and emmeans. Results are presented as mean differences, odds ratios, effect sizes (partial η^2^, Cohen’s d), and 95% confidence intervals.

## 3 Results

Between July 2020 and June 2023, 86 patients with first-ever stroke were screened, of which 43 patients (15 women) were eligible for inclusion (for details, see Supplementary Figure 1, CONSORT flow diagram). As shown in Table 1, the 43 patients had a mean age of 71 years (SD = 9), a mean of 12.9 years (SD = 2.0) of formal education, and a mean global cognition score [MoCA (44)) of 17.8 (SD = 4.1]. On average, patients were 40 days post stroke (SD = 20), had predominantly experienced a moderate ischemic (74%) stroke with a mean NIHSS (National Institutes of Health Stroke Scale) score of 10.7 (SD = 2.6), and the majority (62%) did not present with hemianopia. Patients included in the study were characterized by poor trunk stability (TIS: mean = 7.3, SD = 4.9), a tilted vertical perception (SVV: mean = 11.7, SD = 7.4), and high functional dependency (Barthel Index: mean = 13.3, SD = 16.6). They also reported moderate pain intensity (mean = 5.7, SD = 3.1) on a numeric pain rating scale (single item Global07 of PROMIS-Profile-29 v2.1, PROMIS Health Organization (PHO) (45)), ranging from 0 (no pain) to 10 (worst pain imaginable). All patients were non-ambulatory (FAC 0–2), and the majority of patients were completely unable to walk (FAC = 0, *n* = 23) or required continuous substantial assistance from another person to support body weight, maintain balance, or maintain coordination (FAC = 1, *n* = 13). A smaller group of patients were dependent on walking and needed intermittent support from another person (FAC = 2, *n* = 7).

**Table 1:** Sociodemographic and clinical characteristics for all included patients (*N* = 43) and training groups at baseline.

Characteristics	Overall *N* = 43	Gait training *N* = 25	Standing training *N* = 18	SMD
Age in years, mean (SD)	71.0 (9.4)	71.8 (9.0)	70.0 (10.2)	−0.19
Male patients, n (%)	28 (65%)	18 (72%)	10 (56%)	0.35
Education in years (max. 21)				
Mean (SD)	12.9 (2.0)	13.4 (2.0)	12.4 (2.0)	−0.47
Median [IQR]	12.0 [12.0, 13.0]	13.0 [12.0, 14.0]	12.0 [12.0, 13.0]	
Unknown	8	8	0	
MoCA at baseline				
Mean (SD)	17.8 (4.1)	18.0 (4.3)	17.6 (3.9)	−0.10
Median [IQR]	17.5 [14.0, 20.0]	17.5 [14.0, 21.3]	17.5 [14.5, 20.0]	
Unknown	1	1	0	
Ischemic stroke	32 (74%)	16 (64%)	16 (89%)	0.61
Presence of hemianopsia				0.28
No	26 (62%)	16 (67%)	10 (56%)	
Incomplete	9 (21%)	5 (21%)	4 (22%)	
Complete	7 (17%)	3 (13%)	4 (22%)	
Unknown	1	1	0	
Time from stroke in days at inclusion				
Mean (SD)	40.0 (20.4)	36.5 (19.5)	44.8 (21.3)	0.42
Median [IQR]	34.0 [24.0, 55.0]	31.0 [23.0, 46.0]	40.0 [26.0, 62.0]	
NIHSS at inclusion				
Mean (SD)	10.7 (2.6)	10.8 (2.4)	10.5 (2.8)	−0.12
Median [IQR]	11.0 [9.0, 12.0]	11.0 [9.0, 12.0]	10.0 [9.0, 12.8]	
Barthel index at baseline				
Mean (SD)	13.3 (16.6)	11.8 (16.3)	15.3 (17.2)	0.21
Median [IQR]	5.0 [0.0, 22.5]	5.0 [0.0, 20.0]	7.5 [0.0, 30.0]	
Numeric pain intensity rating scale^a^				
Mean (SD)	5.7 (3.1)	6,0 (2.7)	5.3 (3.8)	0.67
Median [IQR]	6 [3.8,8]	6 [5,8]	7 [1,8]	
Unknown	1	0	1	
FAC at baseline				
Mean (SD)	0.6 (0.8)	0.7 (0.8)	0.6 (0.7)	−0.13
Median [IQR]	0 [0,1]	0 [0,1]	0 [0,1]	
TIS at baseline				
Mean (SD)	7.3 (4.9)	7.1 (5.0)	7.6 (4.8)	0.10
Median [IQR]	8 [4,11]	8 [2.5,10.5]	7.5 [4,11.5]	
SVV at baseline				
Mean (SD)	11.7 (7.4)	12.4 (8.5)	10.8 (5.5)	−0.22
Median [IQR]	10 [7,16]	10 [7,17]	10.5 [6.8,15.3]	

Data were given as absolute frequencies, mean and standard deviation, or median and interquartile range [25th and 75th percentile]. SMD, standardized mean difference; SD, standard deviation; IQR, interquartile range; MoCA, Montreal Cognitive Assessment; NIHSS, National Institutes of Health Stroke Scale; FAC, Functional Ambulation Category; TIS, Trunk Impairment Scale; SVV, subjective visual vertical; max, maximum.

a Item Global07 taken from PROMIS–29 Profile v2.1 (PROMIS Health Organization (PHO); numeric rating scale ranging from 0 (no pain) to 10 (worst pain imaginable)).

The groups were comparable at baseline regarding sociodemographic variables, stroke severity, functional dependency, subjective pain intensity, and global cognition (see Table 1). Of the 43 patients, 25 were assigned to the GT group (low TIS: *n* = 13, moderate TIS: *n* = 12), and 18 were assigned to the ST group (low TIS: *n* = 9, moderate TIS: *n* = 9). Of the 43 patients, 36 completed at least 6 of the 9 interventions and follow-up measurements. All dropouts occurred in the GT group: one patient withdrew consent and 6 withdrew consent shortly after the start of GT.

### 3.1 Safety evaluation

End-effector-based gait training was discontinued in six patients. One patient discontinued after four sessions due to a non-study-related toe injury. Two patients were withdrawn preemptively by the study physician after one and two sessions, respectively, because of pre-existing cardiac conditions (myocardial infarction and congestive heart failure) and signs of exercise intolerance (decreased oxygen saturation). Three patients discontinued due to pain (one after three sessions, one after five sessions), of which two had pre-existing knee pain. No immediate or delayed adverse events occurred. All affected patients were able to continue routine motor therapy. Therefore, these events were not classified as serious adverse events (see Supplementary Table 2).

### 3.2 Primary outcome

**Figure 1:**
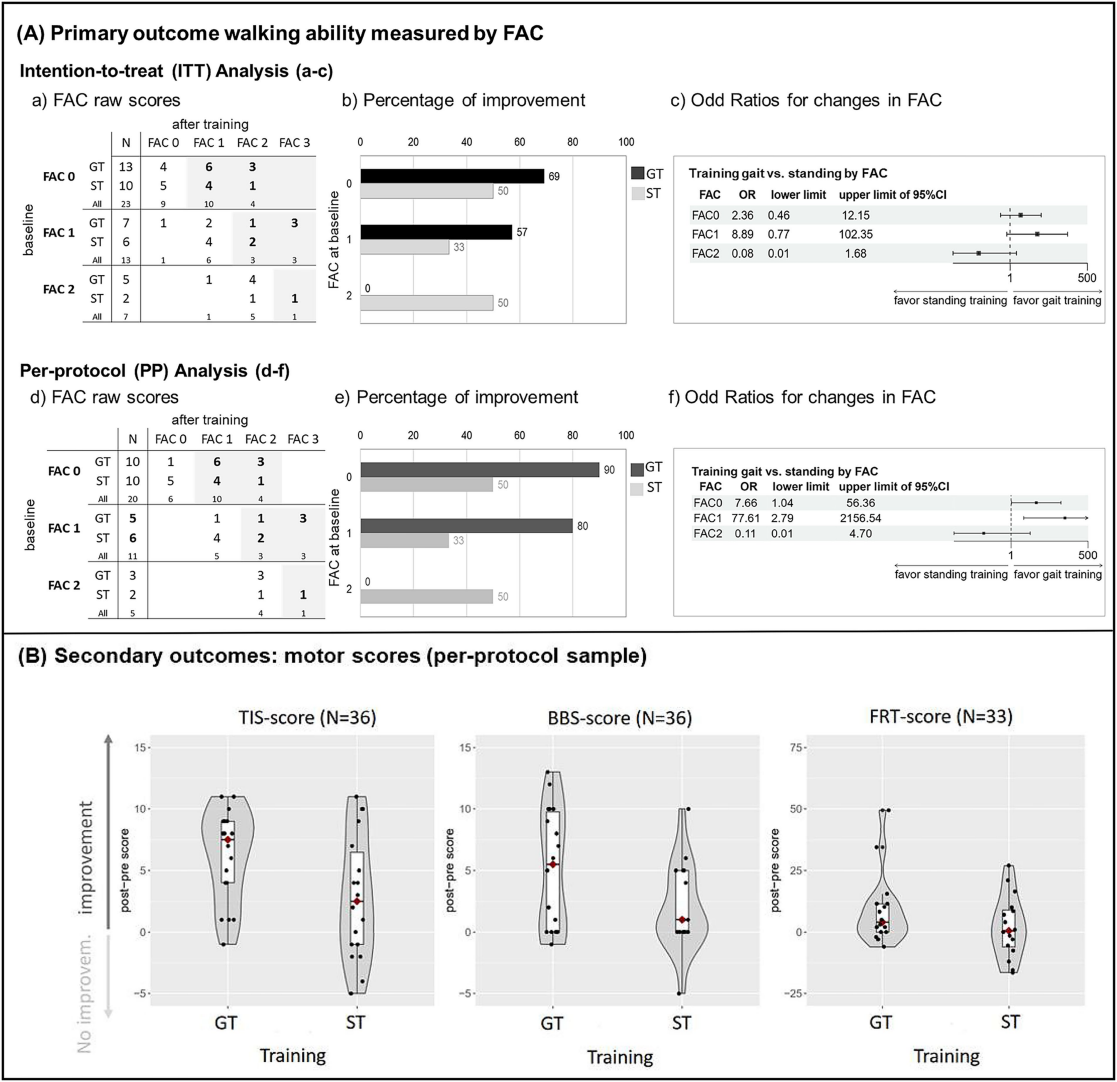
**(A)** Results of the primary outcome functional ambulation category (FAC) in the intention-to-treat sample (*N* = 43), including dropouts; **(a–c)** and the per-protocol sample (*N* = 36), excluding dropouts; **(d–f)**. Left panel: Cross-table of pre- and post-intervention FAC raw scores for gait training (GT) and standing training (ST), with patient frequencies per cell (improvements in FAC scores are highlighted); Center panel: Horizontal bar charts showing the percentage of patients with improved FAC scores after GT and ST, displayed by baseline FAC score; Right panel: Forest plot illustrating marginal estimated effects (proportional odds ordinal regression) of GT vs. ST on post-training FAC scores, with pairwise comparisons across baseline FAC scores. Points represent the mean odds ratio (OR), and horizontal lines represent the 95% confidence interval. An OR of 1 refers to no effect, an OR > 1 favors GT, and an OR < 1 favors ST. **(B)** Combined violin and box plots for the secondary outcomes. Depicted are difference scores (post-pre intervention) for trunk impairment (TIS, *N* = 36), balance (BBS, *N* = 36), and the Functional Reach Test (sitting condition, *N* = 33) separately for the GT and ST groups. Values > 0 indicate “improvement,” values ≤ 0 correspond to “no improvement” or deterioration. The red diamond represents the median of each group. FAC: Functional Ambulation Category, GT: gait training, ST: standing training, OR: odds ratio, TIS: Trunk Impairment Scale, BBS: Berg Balance Scale, FRT: Functional Reach Test.

Analysis was initially performed on the ITT sample (*N* = 43), as shown in Figure 1Aa–c. The analysis was then replicated in the PP sample (*N* = 36), with results presented in Figure 1Aa–f. The Brant test indicated that the proportional odds assumption holds in ordinal regression analyses (omnibus *p* > 0.3). In the ITT analysis, the odds ratio was 1.20 (95% CI [0.30–4.78], *p* = 0.792), indicating no overall superiority of GT across all FAC levels. Consistent with previous findings on repetitive gait training (3), we observed a positive correlation between the number of training sessions performed and FAC gain (Spearman’s rho r = 0.552, *p* = 0.004), which justified conducting a more sensitive PP analysis. In the PP sample, the overall odds ratio (OR) was not significant OR = 4.08 (95% CI [0.80–20.87], *p* = 0.091). However, Figure 1A (panel f) shows a forest plot of the ordinal regression model (training condition as the independent variable, with adjustments for FAC baseline, TIS baseline, amount of concomitant therapy, and an interaction term between FAC baseline and intervention). The forest plot suggests an interaction between baseline FAC and the training group. This implies that any potential benefit of GT was confined to patients with low baseline FAC scores. Figure 1A: panels d-f show, from left to right: baseline FAC distributions were similar between the groups, with most patients classified as FAC 0 (panel d); among patients with low baseline FAC scores (FAC 0–1), a higher proportion improved in the GT group compared to ST (panel e) and as can be seen in the forest plot (panel f), patients with a low baseline FAC score (FAC 0 or 1) had a higher chance of improving FAC after GT compared with ST, whereas patients with baseline FAC 2 did not.

### 3.3 Motor functions (secondary outcomes)

The results are shown in Table 2 and Figure 1B. The findings demonstrate improvements following GT compared to ST in trunk impairment (TIS: mean difference [95% CI] = 3.4 [0.8, 6.1], partial η^2^ = 0.18) and balance (BBS: 5.7 [0.5, 6.7], partial η^2^ = 0.15). Forward-leaning ability differed only slightly between the groups (FRT sitting: mean difference [95% CI] = 4.0 [−0.2, 17.3], partial η^2^ = 0.12). To contextualize the observed changes, we compared the data with available minimal clinically important difference (MCID) values. Clinically relevant changes were more frequently observed in TIS [MCID = 3; (46)] and BBS scores [MCID = 6–7; standard scale (47)] after GT compared to ST. The percentages were 78 and 50% for GT, respectively, compared with 33 and 11% for ST. Changes in FRT sitting scores [MCID = 6 cm; (48)] occurred with similar frequency in both groups (GT = 41%, ST = 38%). Baseline TIS score did not correlate with change in FAC (post-pre difference) for either GT (r = −0.01) or ST (r = −0.09), indicating that baseline TIS plays no substantial role in FAC improvement after training.

**Table 2:** Motor, neglect, subjective visual vertical perception, and mood outcomes (per-protocol sample *N* = 36), with main group comparison results (ANCOVA).

Motor functions	*N*	Gait training (GT)		Standing training (ST)		ANCOVA GT vs. ST		
		Pre	Post	Pre	Post	Mean Diff.	95% CI	Part. η^2^
TIS	36	7.8 (4.8)	13.9 (3.4)	7.6 (4.8)	10.4 (5.5)	3.9	1.3,6.5	0.22
BBS	36	2.2 (3.2)	7.3 (6.3)	2.0 (3.3)	3.5 (4.7)	3.2	0.04,6.3	0.12
FRT (sitting) in cm	33	35.1 (15.5)	42.9 (8.5)	30.8 (14.4)	31.9 (16.1)	8.6	−1.0,17.8	0.11
Visuospatial neglect test								
Bells test: CoC	35	0.25 (0.32)	0.19 (0.25)	0.34 (0.28)	0.23 (0.29)	0.05	−0.07,0.17	0.02
Number of left omissions	35	6.8 (5.3)	5.7 (5.7)	9.6 (5.4)	6.2 (5.3)	2.0	−0.9, 4.9	0.06
LBT	36	2.7 (2.8)	1.2 (2.4)	3.6 (2.9)	2.3 (3.3)	−0.4	−1.4,0.7	0.02
Copy a figure	35	5.2 (2.5)	6.2 (3.5)	4.0 (2.4)	6.3 (2.0)	−0.5	−1.9,0.9	0.02
Saccade position^a^	35	7.7 (7.8)	4.9 (6.9)	11.6 (8.1)	7.2 (7.8)	0.3	−2.9,3.4	<0.01
Other outcomes								
CBS	28	13.7 (6.6)	14.9 (7.6)	14.8 (10.5)	13.4 (8.3)	2.6	−3.2, 8.4	0.04
SVV range	36	11.5 (7.2)	6.1 (3.5)	10.8 (5.5)	6.3 (5.2)	−0.2	−3.2,2.9	<0.01
Anxiety (T-score)^b^	34	49.3 (12.7)	49.0 (12.2)	54.4 (10.5)	52.2 (8.5)	1.6	−4.2,7.5	0.01
Depr. (T-score)^b^	34	43.6 (8.9)	45.7 (9.4)	50.4 (8.4)	49.7 (9.9)	−1.1	−8.6,6.5	<0.01

Pre- and post-training data are given as mean and standard deviation. ANCOVA: Training condition as a between-subject factor, Covariates: trunk stability (stratifying variable), respective baseline score, and amount of concomitant motor therapy. Diff, difference; CI, confidence interval; Part, partial; FAC, Functional Ambulation Category; TIS, Trunk Impairment Scale; BBS, Berg Balance Scale; FRT, Functional Reach Test; CoC, center of cancellation; LBT, Line Bisection Test (deviation in cm); nb, number of left errors; CBS, Catherine Bergego Scale; SVV, subjective visual vertical; Depr, depression.

a Eyemove©.

b Assessed by the State–Trait-Anxiety-Depression Inventory.

### 3.4 Neglect, SVV, and mood

After the intervention, no between-group differences were found in assessments of visuospatial neglect or SVV perception. Both groups showed similar improvements over time, suggesting that the interventions had no differential impact on the recovery of visuospatial neglect symptoms and SVV perception. Neglect-related functional disability (CBS) was also comparable between the groups. Mean Tscores of self-rated mood (anxiety and depression) were within the normal range (T ≤ 60), showed no substantial between-group differences, and remained stable over time. For details, see Table 2.

Comparing the performance of the two training conditions (see Table 3) revealed that: (i) patients spent fewer minutes in GT than in ST over the intervention period (mean difference [95% CI]: −49 [−89, −9], Cohen’s d = 0.8); (ii) patients trained fewer minutes per session in GT than in ST (mean difference [95% CI]: −5 [−9, −1], Cohen’s d = 0.9); (iii) training induced greater objective strain (change in heart rate) after GT than ST (mean difference [95% CI]: 5 [0.1, 9.1], Cohen’s d = 0.8); and (iv) subjective strain ratings (Borg scale) showed almost no difference between groups (mean difference [95% CI]: 0.3 [−0.8, 1.3], Cohen’s d < 0.1). During GT, mean walking speed was lower than the 1.5 km/h specified in the study protocol (mean: 1.3 km/h, SD = 0.2 km/h), and BWS exceeded the predefined maximum of 30% (mean: 32%, SD = 14%). FAC gain (difference score) showed a moderate association with mean walking speed (r = 0.38, *p* = 0.12), but no association with BWS (r = −0.11, *p* = 0.68). Patients in the GT group received slightly more routine motor treatment on average during the intervention period compared with the ST group (mean difference in minutes [95% CI]: 116 [−65,297], Cohen’s d = 0.4). Both groups received a similar amount of neglect treatment (mean difference in minutes [95% CI]: 8 [−78, −95], Cohen’s d = 0.1).

**Table 3:** Process measures and training data (time and settings of gait trainer) per session: Descriptive statistics and group comparison (*t*-test gait vs. standing training).

Process measures	Gait training (GT) *N* = 18		Standing training (ST) *N* = 18		GT vs. ST		
	Mean (SD)	[min, max]	mean (SD)	[min, max]	Mean Diff.	95% CI	Cohen’s d
Numbers of training sessions	9 (0.6)	[7, 9]	9 (0.7)	[6, 9]	−0.1	−0.6,0.3	<0.1
Training time on average (max. 270 min)	165 (48)	[83, 270]	214 (68)	[60, 270]	−49	−89, −9	0.8
Average training time per session (max. 30 min)	20 (5)	[13, 30]	25 (6)	[9, 30]	−5	−9, −1	0.9
Δ Heart frequency, post-pre session in bpm^a^	10 (8)	[−2, 26]	5 (5)	[−6, 16]	5	0.1, 9.1	0.8
Perceived strain during training (Borg scale: 0–20)	14 (2)	[12, 17]	14 (2)	[11, 16]	0.3	−0.8, 1.3	<0.1
Routine motor treatment therapy, minutes	1003 (242)	[540, 1500]	887 (290)	[510, 1530]	116	−65, 297	0.4
Routine neglect treatment therapy, minutes	165 (134)	[30, 420]	157 (120)	[0, 360]	8	−78, 95	0.1

Training time per session (in minutes)									
Session (T)	T1	T2	T3	T4	T5	T6	T7	T8	T9
GT: median [percentile 25,75]	14 [8,15]	16 [11,20]	17 [14,21]	18 [15,22]	19 [15,26]	20 [17,27]	22 [20,30]	20 [20,30]	23 [16,30]
ST: median [percentile 25,75]	29 [11,30]	30 [16,30]	25 [16,30]	30 [16,30]	30 [30,30]	30 [21,30]	30 [29,30]	30 [21,30]	30 [20,30]

Setting gait trainer per session (T)	T1	T2	T3	T4	T5	T6	T7	T8	T9
Walking speed, km/h (min. 1.5 km/h)	1.0 (0.4)	1.2 (0.4)	1.3 (0.4)	1.2 (0.3)	1.3 (0.2)	1.3 (0.2)	1.4 (0.2)	1.5 (0.2)	1.4 (0.2)
Nb. of steps	517 (284)	735 (348)	776 (363)	816 (333)	917 (342)	986 (406)	1162 (371)	1183 (355)	1148 (450)
Body weight support, in % (max. 30%)	33 (13)	33 (12)	34 (14)	33 (14)	31 (16)	31 (16)	30 (17)	29 (16)	28 (16)

Cohen’s d: values 0.2 to 0.4 correspond to a small effect, values 0.5 to 0.8 correspond to a medium effect, and values > 0.8 correspond to a large effect, SD: standard deviation, min: minimum, max: maximum, Diff: difference, CI: confidence interval, T: training, Nb: number, Aver: average, bpm-beats per minute.

a ST: *n* = 17.

## 4 Discussion

To the best of our knowledge, this is the first study to investigate the effects of end-effector-based gait training in severely affected, non-ambulatory patients with VSN after subacute stroke. We compared end-effector-based gait training (GT) plus standard physical therapy with verticalization training using a standing frame (ST) plus standard physical therapy. ST was intended to strengthen the trunk and lower limbs and to support orthostatic regulation as preparation for gait training (49). The odds ratios did not reach statistical significance in either the ITT or PP analysis; therefore, we could not demonstrate overall superiority of GT over ST across all FAC levels. Nevertheless, when considering only patients who completed at least six of the nine interventions, those with severe gait impairment were more likely to improve walking ability with GT than with ST, suggesting that early end-effector-based gait training is feasible and potentially beneficial. In addition, patients in the GT group showed greater improvements in trunk function and balance than in the ST group.

Pain-related dropouts during GT may indicate an increased risk associated with end-effector-based gait training. In one case, GT appeared to exacerbate pre-existing knee pain, but this was not observed in other patients (see Supplementary Table 2). Pain was also common prior to the intervention and in those who continued training, typically resulting from chronic degenerative diseases, severe hemiparesis, shoulder subluxation, inability to self-position, or neglect-related limb injuries. This reduces the likelihood of a systematic dropout bias. For multimorbid and severely affected patients, mobilization in standing and walking is generally strenuous and likely impacts patients’ training tolerance. Additionally, reduced engagement or predispositions such as anxiety may also have contributed to discontinuation.

Although limited to the PP sample, our results suggest beneficial effects of GT compared with ST, particularly in patients with VSN and severe gait impairment (FAC 0–1). This effect was not observed in patients with FAC 2, which may be explained by the very small subgroup size (*n* = 3 in GT; see Figure 1A, left panel). This limitation likely weakened the overall odds ratio and increased the risk of a Type II error. Nevertheless, our findings extend previous evidence supporting end-effector-based gait training in the early post-stroke phase by showing that even patients with severe impairment and concomitant VSN can benefit. This patient group has been underrepresented both in rehabilitation guidelines, such as the German ReMoS guideline (3), and in studies investigating the relationship between trunk training, balance, and gait (50). Our findings are in line with a previous cohort study (51) and a meta-analysis (52), which also reported greater improvements in the ability to walk through GT in severely impaired, subacute, non-ambulatory stroke patients in general.

Although verticalization via ST represents a common practice standard for patients with severe hemiparesis and VSN, its comparability is limited. This may have affected the internal validity of the study. Nevertheless, both ST and GT stimulated orthostatic regulation, muscle strengthening, and trunk stability; ST was therefore chosen as the control condition. More detailed logging of ST parameters in future studies could improve quantification and comparability.

The exploratory analysis indicated that the GT group achieved greater improvements in trunk impairment (TIS) and balance (BBS) than the ST group. While exploratory, these findings are clinically meaningful and relevant for practice. Therefore, GT may potentially enhance both sitting and standing balance, which are essential for regaining mobility (50). Our results are consistent with prior studies in non-ambulatory subacute stroke patients without additional VSN (53). Notably, no correlation was found between initial TIS and improvement in FAC, indicating that the beneficial training effects of early end-effector-based gait training—improved walking ability, balance, and trunk stability—were independent of patients’ initial trunk stability. Even patients with low trunk stability benefited from GT.

Both groups demonstrated similar improvements in VSN symptoms over time. As no neglect-specific interventions were provided in either group, the observed improvements likely reflect spontaneous recovery and recovery resulting from conventional neglect therapy. Importantly, early gait training had no adverse impact on VSN recovery. To more accurately assess the impact on ego- and allocentric neglect subtypes, future studies should incorporate more sensitive measures such as eye tracking or virtual reality.

With respect to perceived exertion, both interventions were tolerated equally well and were rated as “somewhat difficult” on the Borg scale (mean scores: 14.6 vs. 13.8 for GT and ST, respectively). Thus, GT was not perceived as more strenuous than ST.

## 5 Limitations

Our study has several limitations. First, the small sample size led to unstable estimates with wide confidence intervals and increased the risk of a Type II error. Dropouts further reduced statistical power, and due to low recruitment rates and limited resources during the COVID-19 pandemic, we were unable to increase the sample size. Multicenter studies would enable higher recruitment and stratification into sufficiently large, equally sized FAC subgroups, providing more reliable conclusions across different baseline levels.

Second, protocol deviations occurred. The initially pre-specified gait speed proved too ambitious for severely affected patients (see Table 3). Although training parameters could be increased gradually over the sessions, predefined target parameters were only reached toward the end of training. As gait speed was associated with FAC improvement, this may have reduced the treatment’s effect. Nevertheless, these deviations reflect the real-world applicability of the protocol in this severely impaired cohort. Future studies should therefore consider progressively challenging training protocols.

Third, although dropouts may suggest reduced device tolerability, the majority of patients in both groups continued training despite experiencing pain. The reasons for dropout were likely multifactorial and not solely related to attrition bias. Careful pain monitoring may help minimize adverse events and improve adherence.

Finally, the lack of long-term follow-up limits conclusions about sustained functional benefits. Despite this limitation, we demonstrated promising short-term effects in patients with VSN, supporting the feasibility of end-effector-based gait training in this understudied cohort.

## 6 Conclusion

End-effector-based gait training in subacute post-stroke patients with VSN is a feasible adjunct to the standard training option and may improve walking ability. These patients should receive guideline-based post-stroke rehabilitation, including implementation of early end-effector-based gait training (3). Further studies are necessary to determine optimal training duration and intensity, as well as to establish long-term effects. Clinicians should focus on strategies to implement these recommendations into clinical practice.

## Data Availability

The raw data supporting the conclusions of this article will be made available by the authors, without undue reservation.

## Footnotes

^1^ www.drks.de/search/de/trial/DRKS00021654
