# Supplementary material for: Early end-effector-based gait training in non-ambulatory patients with visuospatial neglect after subacute stroke
Source: Front Neurol. 2025 Oct 1;16:1639659. doi: 10.3389/fneur.2025.1639659 (PMC12520930; doi:10.3389/fneur.2025.1639659)
Supplement: Supplementary file 1 [file Table_1.docx]

| Criteria | Gait training  (GT) | Standing training  (ST) | + Motor standard therapy |
| --- | --- | --- | --- |
| Frequency | 3 times per week | 3 times per week | 3 times per week  patient-specific |
| Intensity per session (maximum) | targeted training time: 30 minutes | targeted training time: 30 minutes | 30-45 minutes |
| Treatment duration | 3 weeks | 3 weeks | 3 weeks |
| Conducted by | physiotherapists/ sports therapists | physiotherapists/ sports therapists | physiotherapists/  sports therapists  occupational therapists |
| Program | end-effector supported gait training  THERA-Trainer lyra | early verticalization in a supported standing position  THERA-Trainer balo | tailored to each patient's individual needs;  includes traditional treatment techniques:  -practicing transfers (lying to sitting, bed to wheelchair, sit-to- stand)  - core stability training  - sitting balance  - motor muscle strengthening  exercises  - assisted over-ground walking (if feasable) |
| Support/assistance | targeted body weight support  0-30 % (max.60%) | table unit, patient belt,  knee and pelvic support, forefoot securing system | patient-specific |
|  | targeted walking speed  1.5 km/h | n/a | n/a |
| Documentation | **study protocol**:  date, time, heart rate, number of steps, training duration in minutes,  walking speed in km/h, body weight support in kg, perceived strain  free text: reasons for deviations | **study protocol:**  date, time, heart rate, training duration in minutes,  positioning and aids,  perceived strain  free text: reasons for deviations | routine clinical documentation |
| Shared elements | - verticalization  - orthosthatic activation  - leg and trunk muscle strengthening | |  |

Supplementary Table 1. Description of conducted treatments.
