# Supplementary material for: Early end-effector-based gait training in non-ambulatory patients with visuospatial neglect after subacute stroke
Source: Front Neurol. 2025 Oct 1;16:1639659. doi: 10.3389/fneur.2025.1639659 (PMC12520930; doi:10.3389/fneur.2025.1639659)
Supplement: Supplementary file 2 [file Table_2.docx]

| Patient | Number of sessions (maximum 9) | Dropout initiated by | Reason | Description | Specifically related to GT |
| --- | --- | --- | --- | --- | --- |
| 1 | 0 | patient | withdrawal of consent | n/a | No |
| 2 | 1 | study physian | safety reason | History of congestive heart failure and myocardial infarction. A decrease in oxygen saturation measured by fingertip pulse oximetry occured during gait training | Uncertain;  would probably also occur with other physical exertion |
| 3 | 2 | study physian | safety reason | Pneumonia treated with antibiotics before GT training. Although all blood parameters had returned to normal, the patient showed critical oxygen saturation values during gait training (fingertip pulse oximetry) | Uncertain;  would probably also occur with other physical exertion |
| 4 | 3 | patient | refusal | Since admission this patient was complaining about pain in the left subluxated shoulder, about back pain and about pain caused by chronic arthrosis of the knee joints. The pain symptoms worsened during gait training, so that the training had to be stopped after 3 training sessions. | Probably |
| 5 | 3 | patient | refusal | Showed a post stroke depression and increased anxiety scores. During a self-mobilization not related to our study, the patient fell to ground. The patient did not sustain any injuries, but subsequently refused to participate in the study. | No |
| 6 | 4 | patient | refusal | Aquired a toe injury during inpatient stay which was not related to the GT training. Due to the foot injury, the patient was no longer able to put on shoes and take part in gait training. | No |
| 7 | 5 | patient | refusal | Had been complaining of intermittend knee pain for weeks before gait training. Since an X-ray and clinical examination of the knee joints showed normal findings, the patient was included in the study. The pain persisted and GT training had to be stopped after 5 sessions. | Probably |

Supplementary Table 2. Description of patient dropout and safety evaluation.
