# Supplementary material for: Early end-effector-based gait training in non-ambulatory patients with visuospatial neglect after subacute stroke
Source: Front Neurol. 2025 Oct 1;16:1639659. doi: 10.3389/fneur.2025.1639659 (PMC12520930; doi:10.3389/fneur.2025.1639659)
Supplement: Supplementary file 3 [file Data_Sheet_1.pdf]

CONSORT 2025 Flow Diagram https://www.consort-spirit.org/

Hopewell S, Chan AW, Collins GS, Hróbjartsson A, Moher D, Schulz KF, et al. CONSORT 2025 Statement: updated guideline for reporting randomised trials. BMJ. 2025; 388:e081123. https://dx.doi.org/10.1136/bmj-2024-081123

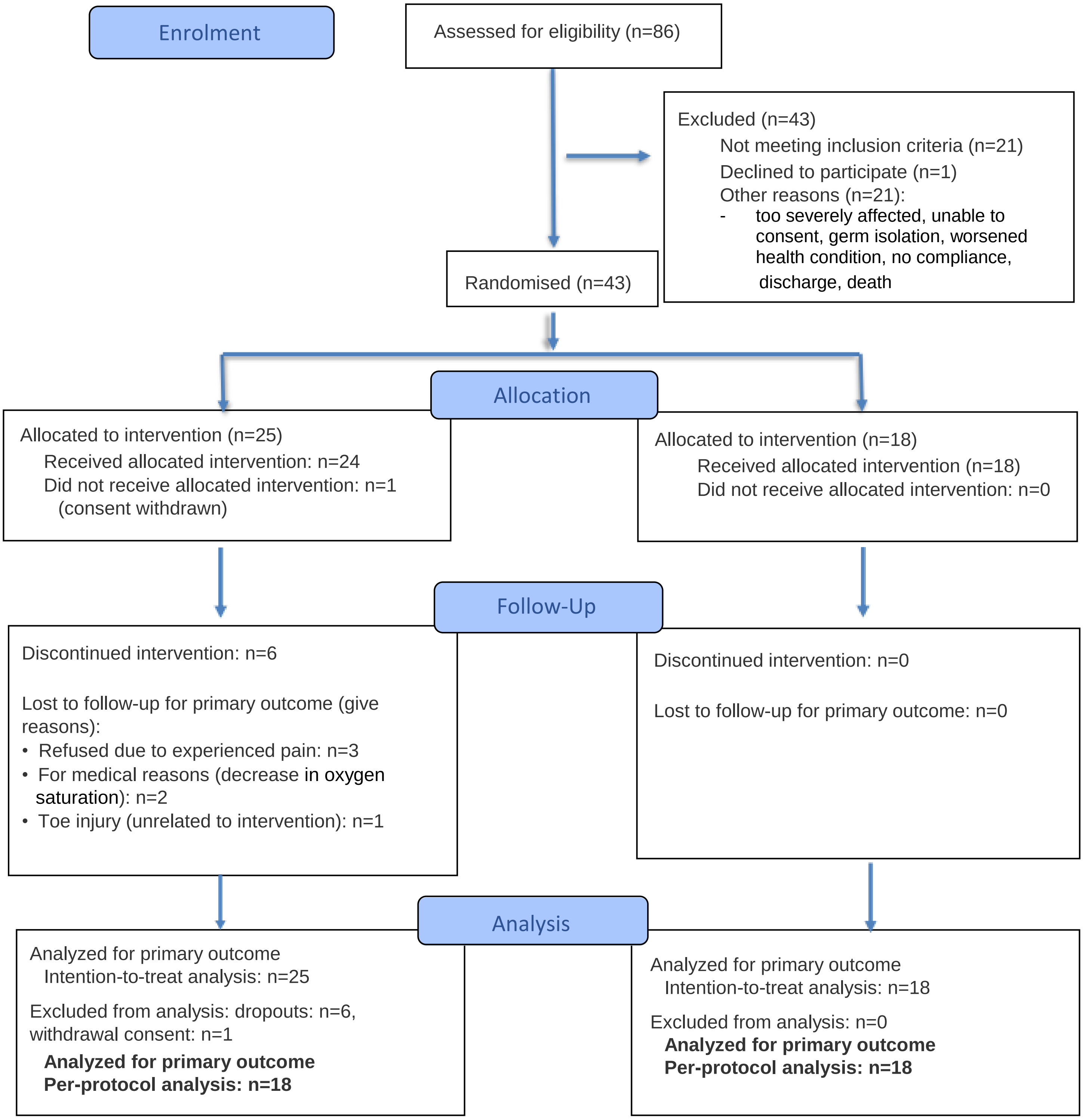

Supplementary Figure 1. Flow chart of the study. Eighty-six eligible patients were enrolled, and 43 consented to participate. Before randomization, patients were classified into two strata (not shown in the flow chart) according to their Trunk Impairment Scale (TIS) score: severely affected trunk function (stratum 1: TIS ≤ 8) or moderately affected trunk function (stratum 2: TIS > 8). Twenty- five patients were allocated to a 3-week end-effector-based gait training program, and 18 patients to 3-week standing training program. In the gait training group, one patient refused to participate before the start of the intervention. Six patients dropped out, completing less than 60 % of gait training and having no subsequent follow-up visit, which was performed shortly after the end of training. Reasons for dropout included pain (n = 3), medical problems (decrease in oxygen saturation during gait training in two patients with pre-existing cardiac risk, n = 2) or another reason (acquired toe injury unrelated to the study, n = 1). Thus, 43 patients were included in the intention-to-treat analysis, and 36 patients were included in the per-protocol (PP) analysis.
